# HEALTH AND WELL-BEING OF OLDER ADULTS IN LONG-TERM CARE FACILITIES: LESSONS FROM LONGITUDINAL STUDIES.

**DOI:** 10.1093/geroni/igz038.907

**Published:** 2019-11-08

**Authors:** Robert O Barker, Andrew Kingston, Fiona Matthews, Barbara Hanratty

**Affiliations:** 1 Newcastle University, Newcastle upon Tyne, United Kingdom; 2 Newcastle University, Newcastle upon Tyne, England, United Kingdom

## Abstract

Older adults in long-term care facilities (LTCF) have complex needs for health care and support. There is a perception that residents’ needs are increasing over time, but little research evidence to back this up. In this study we brought together data on 1640 residents in LTCFs from three longitudinal studies, and conducted repeated cross-sectional analyses across a 25 year period. We found that the prevalence of severe disability amongst residents has increased from 56% to 80% over a 20 year period, driven by increases in difficulties in bathing and dressing. The prevalence of multimorbidity also increased from 29% to 56% between 2006 and 2014. A growth in the number of people with dementia, cardiovascular and cerebrovascular diseases contributed to this. We conclude that residents in LTCFs have become a selected subset of the population, characterised by increasing needs for support. This poses an important challenge for future care provision.

